# Supplementary material for: Loss of MLL3/4 decouples enhancer H3K4 monomethylation, H3K27 acetylation, and gene activation during embryonic stem cell differentiation
Source: Genome Biol. 2023 Mar 3;24:41. doi: 10.1186/s13059-023-02883-3 (PMC9983171; doi:10.1186/s13059-023-02883-3)
Supplement: Supplementary file 1 — Additional file 1: Supplemental Figures. Data and analysis supporting claims made in the manuscript. [file 13059_2023_2883_MOESM1_ESM.pdf]

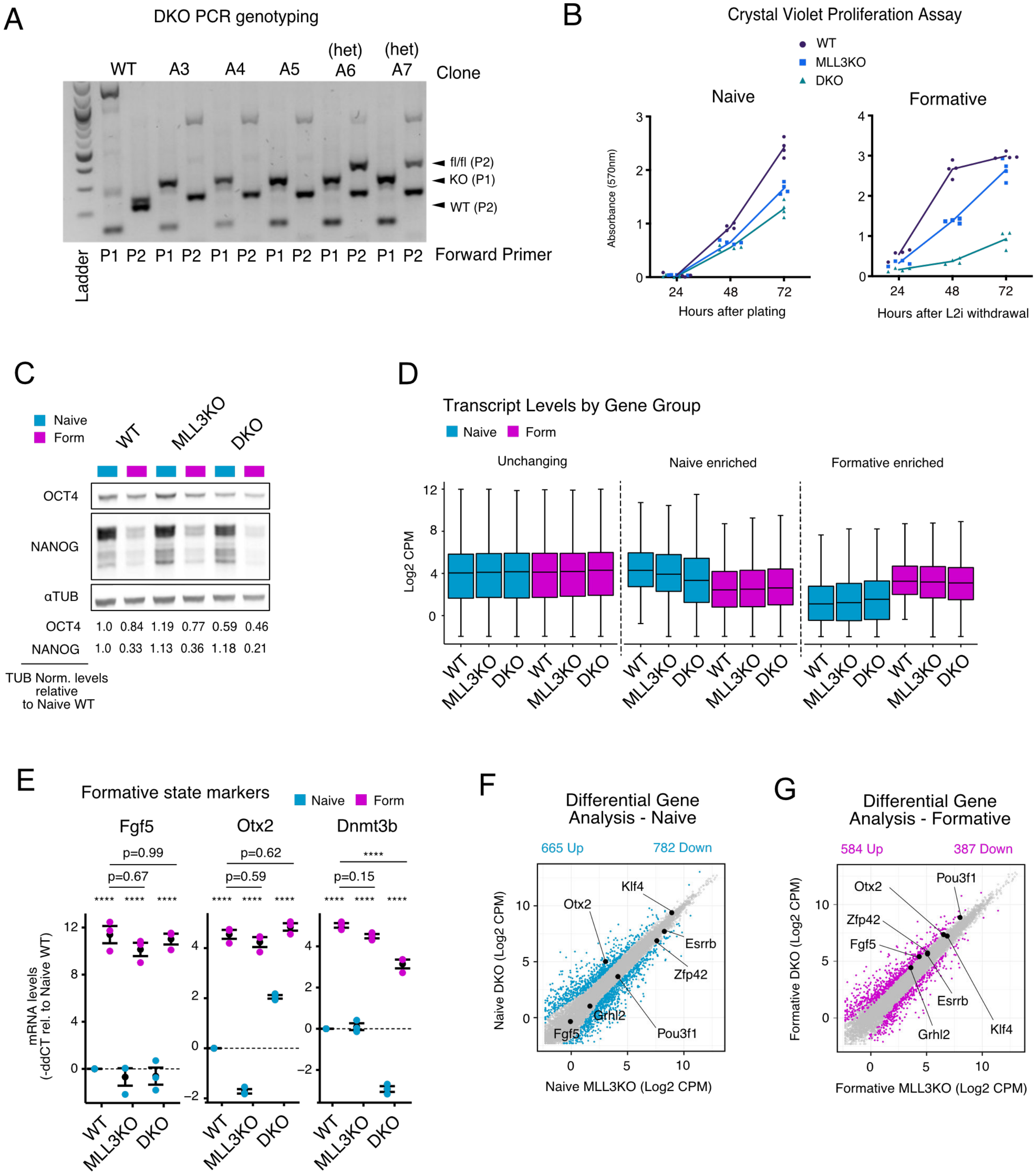

Figure S1: MLL3/4 is dispensable for transcriptional activation of much of the formative program. A) Confirmation of multiple DKO clones using genomic PCR following tamoxifen treatment. B) Proliferation measurements using crystal violet assays. C) Westerns on whole cell protein fractions of OCT4, NANOG and TUBULIN loading control. Quantifications below are fold changes relative to naïve WT after normalizing OCT4 or NANOG for TUBULIN levels in each lane. D) qPCR of formative enriched markers Fgf5, Otx2, Dnmt3b. For each marker selected comparisons are shown resulting from a Two-way ANOVA, Tukey's multiple comparison test for all samples. Mean and S.E.M. in black. Additional statistics provided in supplemental table. E) Boxplots of transcript levels in Log2CPM for unchanging, naïve enriched or formative enriched genes. F) DGE analysis on MLL3KO naive and DKO naive samples (significant genes colored, P.adj < 0.05 and Log2FC > 1). G) Same as F but comparing the formative state. *p<0.05 **p<0.01 ***p<0.001 ****p<0.0001

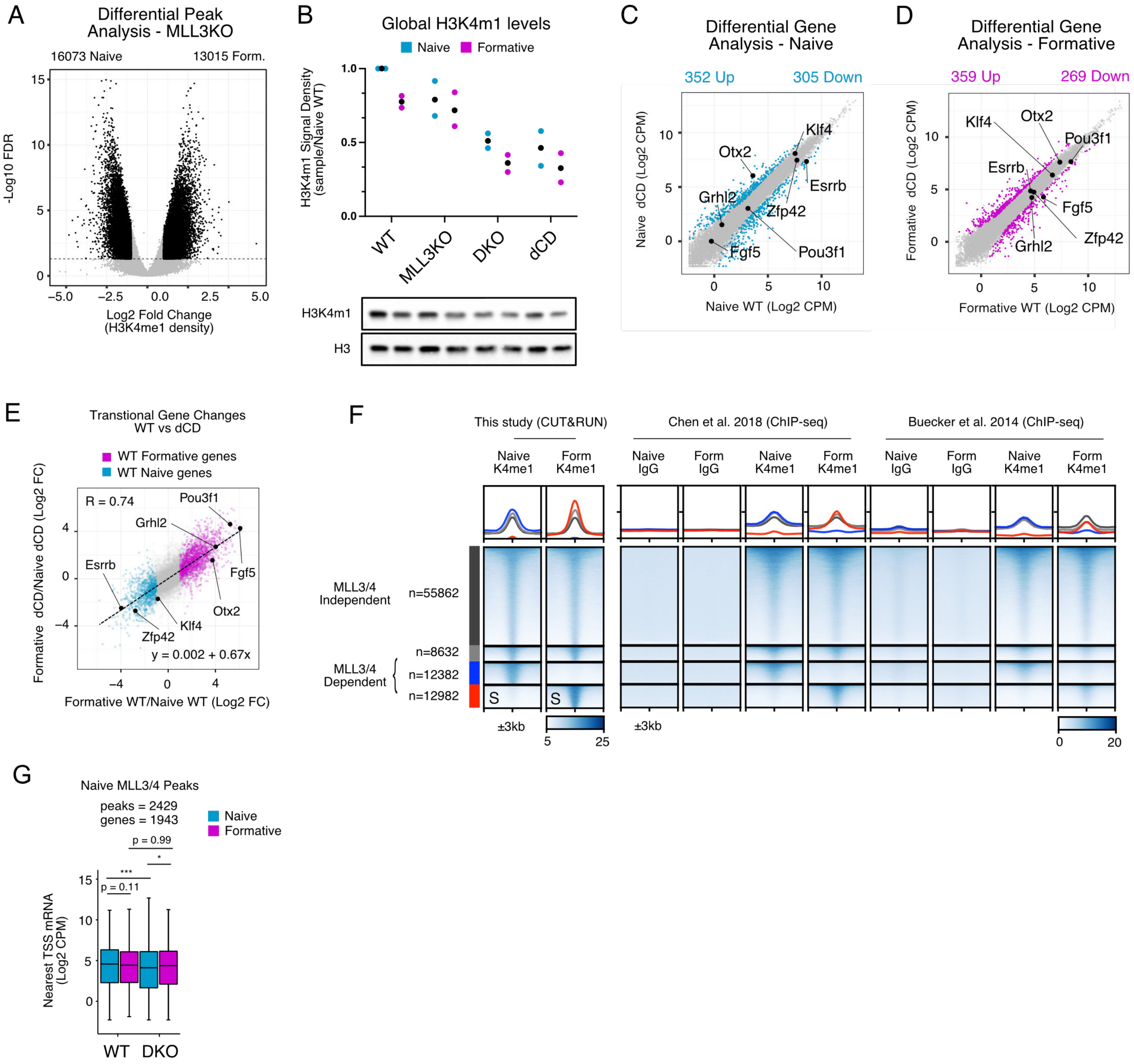

Figure S2: MLL3/4 is required for all dynamic H3K4me1 deposition during pluripotent transition. A) Diffbind analysis of H3K4me1 signal in MLL3KO cells at WT peaks. B) Quantifications of westerns on histones purified by acid-extraction. H3K4me1 levels are relative to naïve WT levels after first normalizing by H3 signal in each lane. Black dot represents mean (n=2). C) mRNA Log2CPM of naive MLL3/4 dCD cells vs. WT cells. Significant genes highlighted. D) same as B with formative samples. E) Foldchange (formative/naive) of all genes for MLL3/4 dCD compared to WT. WT formative and naive genes from WT DGE analysis colored. R, Pearson's coefficient. Dashed line and linear equation represent linear model of all genes. F) Heatmap of ChIP-seq for H3K4me1 in naive and formative cells from two published studies. All heatmap values and range are in CPM. For metagene analysis the range in CPM is the same as shown in heatmap for each factor. G) Nearest neighbor TSS analysis of expression levels in Log2CPM for each RNA-seq dataset near naive MLL3/4 ChIP-seq peak categories. Multi-comparison paired Wilcoxon Rank-Sum Test, Benjamini-Hochberg corrected. *$p<0.05$ **$p<0.01$ ***$p<0.001$ ****$p<0.0001$

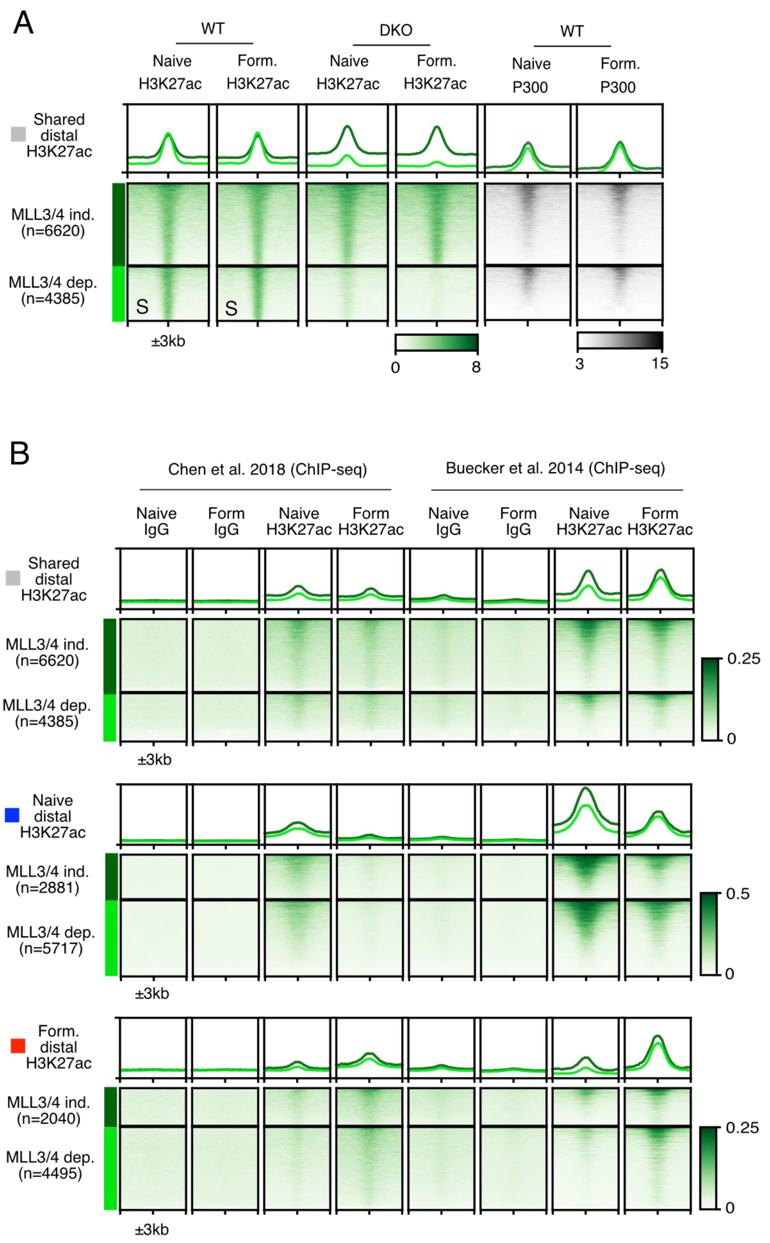

Figure S3: MLL3/4 dependent and independent distal H3K27ac deposition. A) Heatmap of shared H3K27ac sites clustered by MLL3/4 independent or dependent H3K27ac. B) Heatmaps of ChIP-seq for H3K27ac in naive and formative cells from two published studies. All heatmap values and range are in CPM. For metagene analysis the range in CPM is the same as shown in heatmap for each factor.

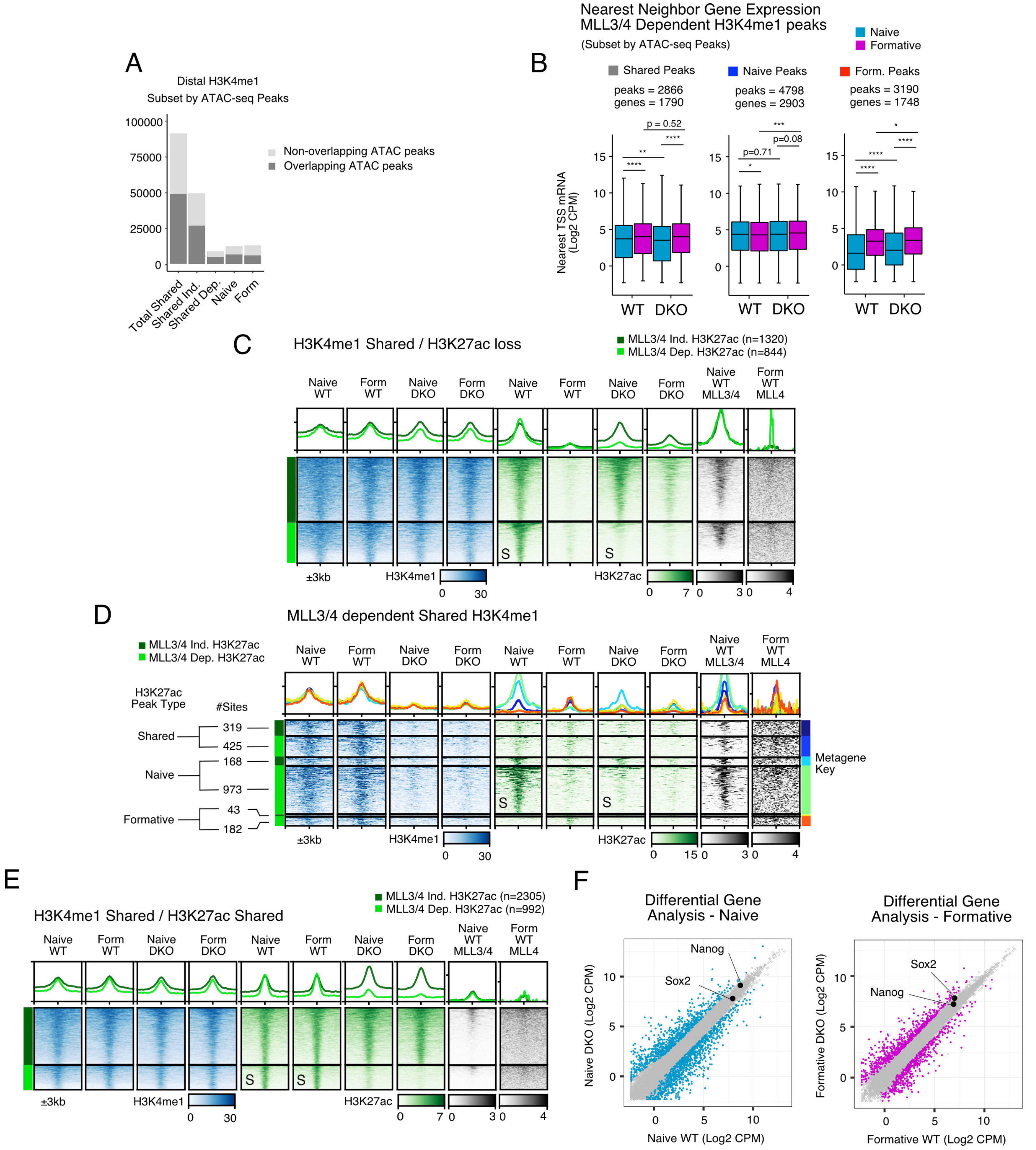

Figure S4: Enhancer Activation Can Occur Independently of MLL3/4. A) Peak count of H3K4me1 categories from Fig2. after filtering for distal H3K4me1 overlapping WT ATAC peaks. B) Nearest neighbor TSS analysis of expression levels in Log2CPM for each RNA-seq dataset near H3K4me1 peak categories after subsetting for ATAC peak overlap. Multi-comparison paired Wilcoxon Rank-Sum Test, Benjamini-Hochberg corrected. C) Heatmaps for shared H3K4me1 that overlaps with naive enriched H3K27ac, clustered by MLL3/4 independent or dependent H3K27ac. D) Heatmaps for MLL3/4 dependent H3K4me1 overlapped with sites that have shared, naive, or formative H3K27ac. Clustered additionally by MLL3/4 independent or dependent H3K27ac. E) Heatmaps for shared H3K4me1 that overlaps with shared H3K27ac, clustered by MLL3/4 independent or dependent H3K27ac. All heatmap values and range are in CPM. For metagene analysis the range in CPM is the same as shown in heatmap for each factor. F) Log2CPM of Sox2 and Nanog in naive and formative samples comparing WT and DKO cells. Sox2 is significantly up in the formative state. *p<0.05 **p<0.01 ***p<0.001 ****p<0.0001

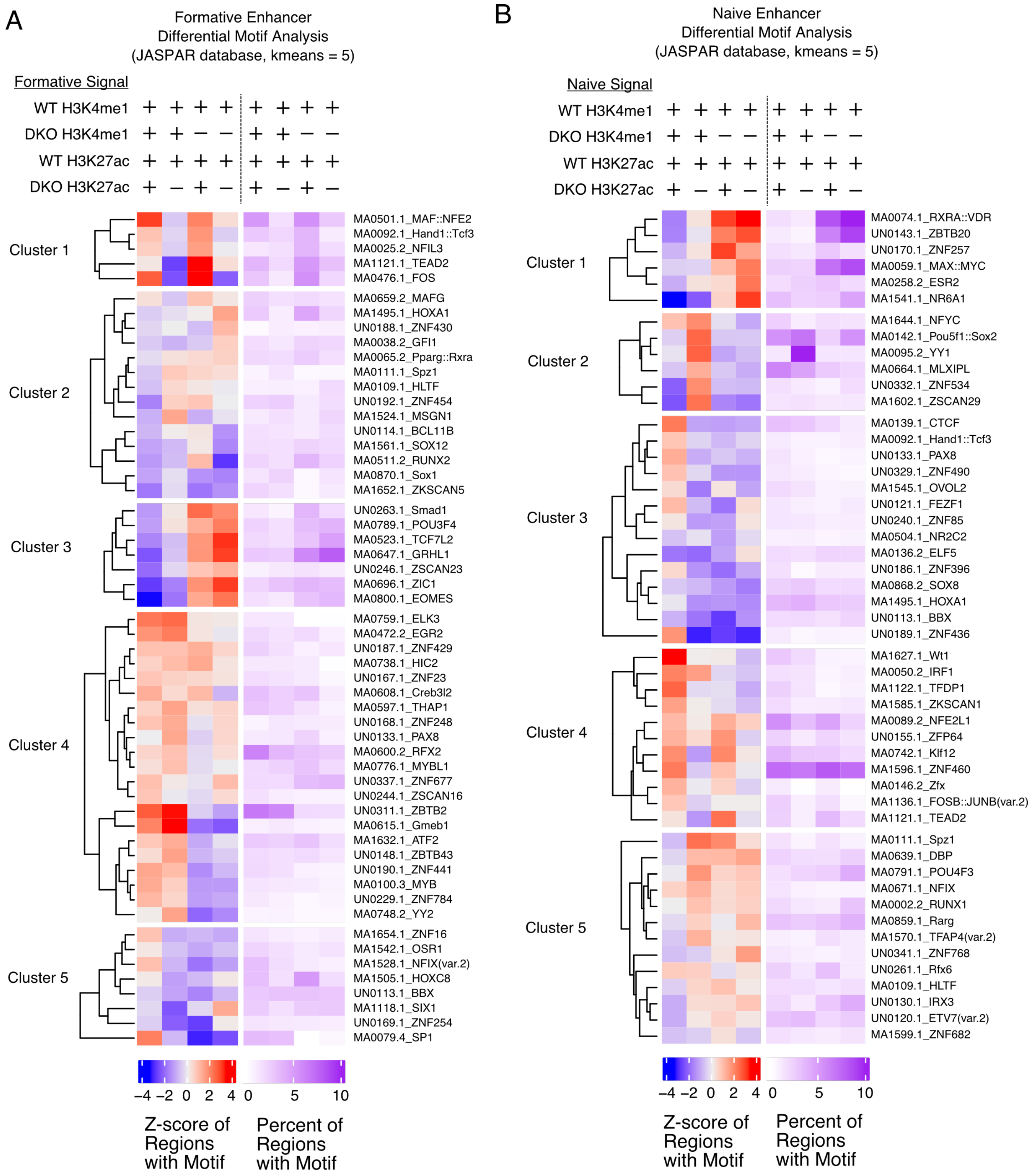

Figure S5: Enhancer Activation Can Occur Independently of MLL3/4. A) Clusters from differential motif analysis using Gimmemotifs on formative enriched MLL3/4 independent and dependent enhancers marked by both H3K4me1 and H3K27ac (Sites from Figure 4C and 4D). Motifs derived from JASPAR 2020 database, k-means clustering used. B) Same as A but with naive enriched independent and dependent enhancers (using sites from Figure 4B and Figure S5B).

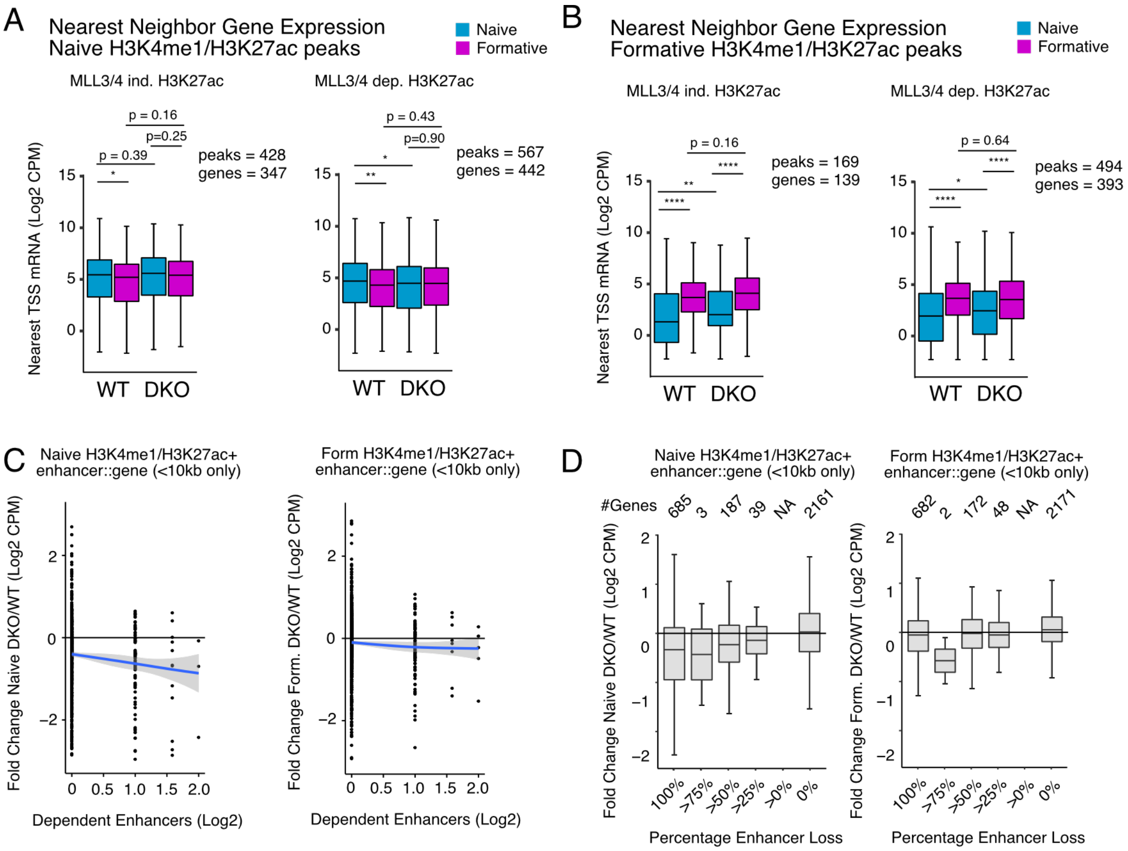

Figure S6: Distal H3K4me1 and H3K27ac are not functionally coupled with formative transcriptional activation. A) Nearest neighbor TSS analysis of expression levels in Log2CPM near naive H3K4me1 dependent sites that are either have MLL3/4 independent (left panel) or dependent H3K27ac (right panel)(clusters from Fig.4B). Multi-comparison paired Wilcoxon Rank-Sum Test, Benjamini-Hochberg corrected. B) Same as A but at formative H3K4me1 dependent sites (clusters from Fig.4C). C) The relative expression DKO/WT of RNA levels for all genes associated with any H3K4me1/H3K27ac+ peak in either naive or formative state compared with the number of dependent enhancers for each gene. Only dependent enhancers within 10kb of the nearest TSS are considered. Each dot represents one gene. Blue line represents generalized linear model, gray 95% confidence interval. D) Boxplots of relative expression DKO/WT of RNA levels for all genes associated with any H3K4me1/H3K27ac+ peak in either the naïve (left) or formative state (right). Each gene is binned by the percentage of their associated enhancer loss in DKOs using only enhancers within 10kb of the nearest TSS. *p<0.05 **p<0.01 ***p<0.001 ****p<0.0001

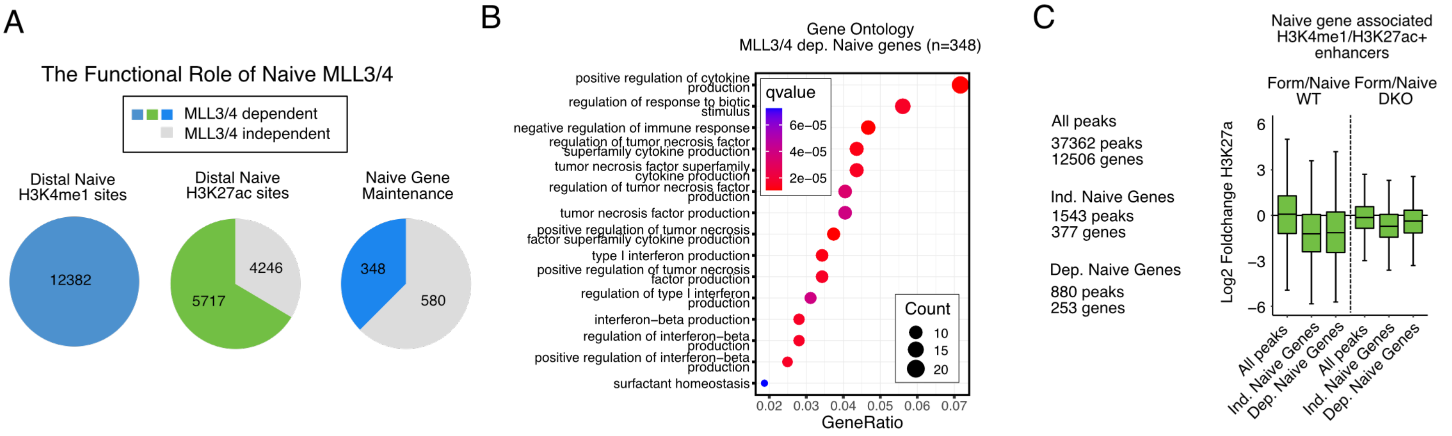

Figure S7: Gene-centric analysis reveals a subset of distal loci associate with MLL3/4 dependent formative genes. A) A major percentage of naive enriched peaks fail to maintain distal H3K4me1 and H3K27ac with moderate consequences on naive gene expression. B) Clusterprofile analysis of Biological Processes Gene Ontology for 348 MLL3/4 dependent naive genes. C) Fold change Log2CPM of H3K27ac density for H3K4me1/H3K27ac+ enhancers that are associated with naive genes whose expression in the naive state is either MLL3/4 independent or dependent. Only a subset of genes in each category were able to be associated with an H3K4me1/H3K27ac+ enhancer.
