# Supplementary figures and images for: Loss of MLL3/4 decouples enhancer H3K4 monomethylation, H3K27 acetylation, and gene activation during embryonic stem cell differentiation
Source: Genome Biol. 2023 Mar 3;24:41. doi: 10.1186/s13059-023-02883-3 (PMC9983171; doi:10.1186/s13059-023-02883-3)

# Additional File 4

Main Fig.1B

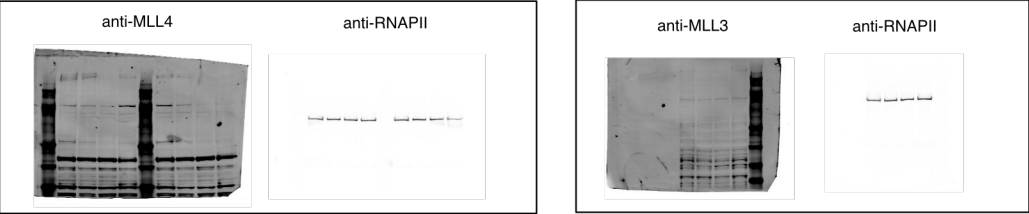

Additional File 1 - Fig.S1C

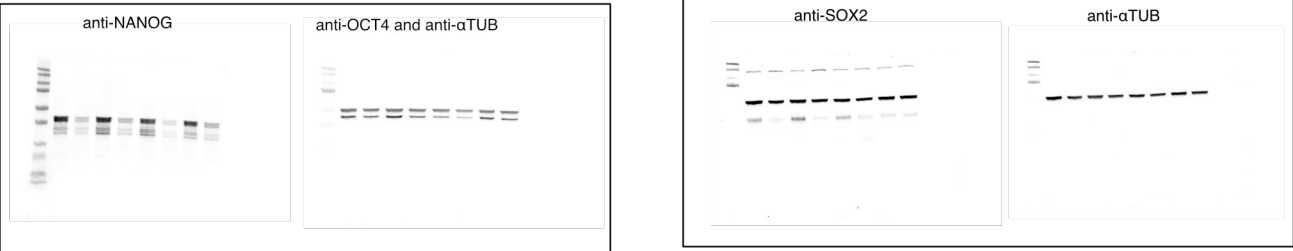

Additional File 1 - Fig.S2B

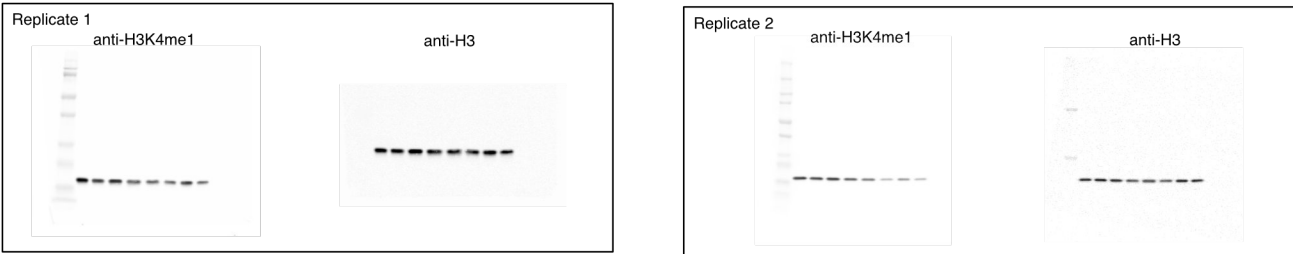

Supplement: Supplementary file 4 — Additional file 4. Western blots. Uncropped images for western blots in Fig. 1B, Additional file 1: Fig. S1C, and Additional file 1: Fig.S2B. [file 13059_2023_2883_MOESM4_ESM.pdf]
