# Supplementary material for: Loss of MLL3/4 decouples enhancer H3K4 monomethylation, H3K27 acetylation, and gene activation during embryonic stem cell differentiation
Source: Genome Biol. 2023 Mar 3;24:41. doi: 10.1186/s13059-023-02883-3 (PMC9983171; doi:10.1186/s13059-023-02883-3)
Supplement: Supplementary file 5 — Additional file 5. Review history. [file 13059_2023_2883_MOESM5_ESM.docx]

Review History

**First round of review**

**Reviewer 1**

**Were you able to assess all statistics in the manuscript, including the appropriateness of statistical tests used?**

No, I do not feel adequately qualified to assess the statistics.

**Comments to author:**

In the manuscript "Loss of MLL3/4 decouples enhancer H3K4 monomethylation, H3K27ac, and gene activation during ESC differentiation" the authors explore the effect that the Mll3/4 have on gene expression during the exit from naïve pluripotency. In previous work it was shown that loss of the catalytic activity of Mll3/4 does lead to loss of monomethylation of Histone H3 on lysine 4. This specific histone modification has been identified as one mark that can be found at enhancers, and it was previously suggested that this monomethylation is used as a recognition for p300 to acetylate the surrounding histones. Here, the authors tested whether such a causative function is true by mapping changes in H3K4me1, H3K27ac and gene expression during the exit from naïve pluripotency. The authors demonstrate that there is no obvious causal relationship between the two histone modifications and that nearby gene expression changes do not necessarily correlate well with these marks.

Overall, this study explores an important question: are these histone modifications that are widely used for the identification of putative enhancer elements causative for gene expression changes. The authors combine differentiation assays with kKnocking out studies, and different genomics assays to then bioinformatically dissect the contributions. The final conclusions are overall negative: the authors do not find that H3K4me1 is a prerequisite for p300 binding or H3K27ac deposition. Furthermore, gene activation is not directly correlated to loss of H3K4me1 or H3K27ac. Therefore, this study expands on previous work from other labs that have identified that H3K4me1 is not needed for the maintenance of the naïve pluripotent state or that H3K27ac is needed for changes in gene expression. The results are strongly supported by the data and the analysis and I do not see any major weaknesses in the study. The main question therefore concerns the novelty of the fundings. Here, I do think that the study might not be the most surprising results based on the above mentioned previous results, but it does very convincingly show that histone modifications are not the main driver of gene expression changes, results that should be presented in this way more often. However, a recent preprint paper(https://doi.org/10.1101/2022.07.18.500459), reported that H2B acetylation, which is specifically catalyzed by CBP/p300, proficiently marks distal enhancers and outperform other methods to predict their target genes. So H2B acetylation will be a perfect marker for enhancer strength. It would be nice to include this in the discussion part.

Minor points:

- The authors compare Mll3Ko, Mll3/4 DKO and WT with each other. It is well described how the single and the double KO are related to each other, however, it is unclear how the WT relates to the KO strains. Is it the parental strain for the single KO? This should be clarified.

- Fig S1B: Labelling issue: in the text it says that the DKO grow slower, but the figure shows that the Mll3KO grows slower compared to the W or the DKO. Which one is correct?

- Similar to the previous comment: the color coding on the supplemental figures is not always clear: the DKO is sometimes orange and sometimes pink.

- Figure 2H and after: what does the "Others" mean and how were they separated from "promoters" and "Distal elements"

- Figure 2I and 3F: the dependent should be removed from the figure, the authors show both, dependent and independent modifications.

- Figure 6A /B/C: it is unclear how the numbers of enhancers related here to each other: in Figure A the authors identify 251 dependent genes, in Figure 6B this number changes to 215 and in Figure 6C to 131. How are these numbers related to each other?

-

- Figure S2A: Labelling issue: in the main text(line number 183), it should be fig.S2A, but labeled as fig.S1A.

- Figure S3B, the citation of Buecker et al.2017, should be Buecker et al.2014.

- For figure 3I, the description and discussion of this figure from line 254 to 259 are not very clear in the main text. Please polish this part.

- Figure 5E, the information of the metric is too short and is not mentioned in the method part. and please add more detailed discription of Figure 5E and F.

**Reviewer 2**

**Were you able to assess all statistics in the manuscript, including the appropriateness of statistical tests used?**

No, I do not feel adequately qualified to assess the statistics.

**Comments to author:**

This manuscript from Boileau et al reports genomic assessment of MLL3/4 KO ESCs under naïve and formative culture conditions. The goal of the work is to determine how MLL3/4, histone methyltransferases that install H3K4me1 at regulatory elements, influence enhancer activity under steady state and during cell state transition. The canonical view is that MLL3/4 recruitment to regulatory elements is upstream of CBP/p300 recruitment and subsequent H3K27ac and gene activation. A number of studies in recent years have suggested that this view is perhaps an oversimplification. Most relevant to this work are two studies from 2017 (PMID 28967912 and 28483418). Two other studies, PMID 29313530 and 29255264, are also relevant but not cited. The conclusions of the submitted work are that (1) many sites of H3K4me1 and downstream H3K27ac are independent of MLL3/4, and (2) loss of MLL3/4 has little effect on the ability of cells to initiate new transcription programs. While the authors provide a quite comprehensive analysis, many of these same conclusions already exist in the current literature.

1. Regarding the growth / morphology of the DKO ESCs, the authors minimize the growth / morphology defects of the DKO (lines 127-136). I would say that the morphology differences as shown are substantial, given that the cells are cultured in 2i. The DKO colony shown is very much different from the WT and MLL3KO. Also, the authors state that the DKO grow at a "slightly slower rate" shown in Fig. S1B. The labels on that figure indicate that MLL3KO are the slowest growing cells. Also, the growth rate of the cells labeled in green seems to be significantly different than that of the other two cell lines, so I don't think it's fair to minimize this as a "slightly slower rate". Related. The "similar levels of OCT4 and NANOG" by WB actually do look lower in the DKO cells (hard to tell / quantify with this assay), which would be in line with the reduced pluripotency evidenced by morphology.

2. I disagree with the interpretation that "gene regulation is largely maintained in the absence of MLL3/4", with a "slight dampening of gene expression" and a "relatively normal" transition from naïve to formative in the DKO - lines 145-152. By eye, it seems like the MLL3/4 DKO both fail to upregulate formative genes and downregulate naïve genes to the same extent observed from the WT, by about 4-fold. This isn't necessarily an inconsequential effect on gene expression, particularly when considering the scope of the defects as shown in Fig. 1G.

3. It would be helpful to define the metrics for differential expression in the text in paragraph starting on line 153. Also, in this analysis, I'd like to see box plot or comparative density plot for expression of naïve enriched, formative enriched, and unchanged in WT and DKO cells under both naïve and formative culture conditions.

4. The authors state the purpose of determining whether gene expression changes were due to direct or indirect effects of MLL3/4 loss (line 169-170) and proceed to describe H3K4me1 genomics. If they want to determine effects of HMT loss, it would be better to focus this analysis based on MLL3/4 enrichment.

5. Line 187 - the authors report few transcriptional differences between the catalytic dead mutants and wild type cells. When coupled with the larger transcriptional disruptions observed in the DKO cells, this finding suggests that loss of the MLL3/4 proteins is more detrimental than loss of catalytic activity. This is the conclusion reached previously by Wysocka and Shilatifard.

6. The authors are reporting that ~2/3 of the H3K4me1 sites of enrichment are independent of MLL3/4. Does this agree with western blot or proteomics analysis of H3K4me1 levels in the DKO and catalytic dead mutant cell lines? Does this agree with previous data sets published by either Wysocka or Shilatifard?

7. The authors report that MLL3/4 loss has no effect on transcription of the dependent naïve peaks. This is different than the conclusion reached by Wysocka, where MLL3/4 loss had a distance-dependent effect on nearest neighboring genes. Can the authors explain the discrepancy?

8. Line 201 - authors state that MLL3/4 ChIP-seq data show enrichment at H3K4me1 sites whether naïve-enriched or shared, but based on the average profile the level of enrichment is much lower at the shared group, and based on the heat map, many of the shared peaks do not appear enriched for MLL3/4. This makes me wonder whether these non-MLL3/4 bound H3K4me1 peaks are real (and therefore should be detectable in antibody-independent proteomics assays, see point 6 above) or whether they are some noise inherent to H3K4me1 genomics.

9. What enzyme is responsible for non-MLL3/4 H3K4me1?

10. Line 223 - the authors state that they intersected H3K27ac C&T peaks with ATAC peaks to enrich for active enhancers. One - wouldn't this also include promoters, or were they computationally excluded? Two - why didn't they perform this additional filter with the H3K4me1 data to identify active regulatory elements? Given the spread or breadth of H3K4me1 peaks, such analysis might change the interpretation of effects on nearest neighboring gene expression shown in the first two figures.

11. Regarding Figure 3, have the authors done similar experiments in the catalytic dead cells to determine whether the MLL3/4-dependent regions require enzymatic activity or simply the protein itself for proper H3K27ac?

12. Line 347 - I find this conclusion confusing, that "only a small number of TFs is dependent on MLL3/4 for maintaining and/or establishing cell state specific H3K4me1 independent of H3K27ac" based on motif analysis only.

13. Line 375 RE enhancer redundancy. The authors report a method by which they attempt to normalize for potential enhancer redundancy. I am not certain that this method (multiplying the number of nearby enhancers for each gene by the number of MLL3/4 dependent acetylation sites) is meaningful in the way that the authors would like it to be.

14. The overall conclusion that I get is that MLL3/4 is important for maintaining cell state but is not important for cell state transitions. This is in contrast to work from the Ge lab and should be discussed.

**Authors Response**

**Point-by-point responses to the reviewers’ comments:**

*Reply: A general concern from both reviewers is a failure on our part to fully cite and distinguish our work from prior publications.*

*Reviewer 1 states: “Therefore, this study expands on previous work from other labs that have identified that H3K4me1 is not needed for the maintenance of the naïve pluripotent state or that H3K27ac is needed for changes in gene expression. The results are strongly supported by the data and the analysis and I do not see any major weaknesses in the study. The main question therefore concerns the novelty of the fundings. Here, I do think that the study might not be the most surprising results based on the above mentioned previous results, but it does very convincingly show that histone modifications are not the main driver of gene expression changes, results that should be presented in this way more often. However, a recent preprint paper(https://doi.org/10.1101/2022.07.18.500459), reported that H2B acetylation, which is specifically catalyzed by CBP/p300, proficiently marks distal enhancers and outperform other methods to predict their target genes. So H2B acetylation will be a perfect marker for enhancer strength. It would be nice to include this in the discussion part.”*

*In terms of “H3K4me1 is not needed in maintenance of naïve pluripotent state”, we assume reviewer 1 is referring to Dorighi et al. 2017 and et al. Rickels 2017. These very important papers show that the catalytic activities of the MLL3/4 are not required in maintenance of gene expression in self-renewing pluripotent cells. Another very recent paper on Biorxiv (https://www.biorxiv.org/content/10.1101/2020.09.14.296558v2) has similarly shown it to be the case with ES cells differentiating toward embryoid bodies. It is important to note, while we use the catalytic mutants as a control in Fig. 2 and show that lack of expression changes during the naïve to formative transition in supplemental Fig. 2, these results are not meant to be a significant component of our story, although we thought it an interesting addition that no effects were seen with differentiation (which is now also supported by the Biorxiv paper). We now reference the Biorxiv paper and further clarify the contribution of the previous work. Importantly, we do not think this takes away from the novelty of our work which centers around the loss of the proteins themselves. As for H3K27ac, we assume the reviewers are referring to the recent paper of Sankar et al. 2020 showing that mutation of the K27 residue such that it cannot be acetylated has little effect on transcription, certainly an exciting and surprising finding. However, it is not directly applicable to our findings. We are showing that even when MLL3/4 loss results in the failure to gain both H3K4me1 and H3K27ac, nearby gene expression is largely unaffected. We think of the marks as surrogates for the presence of the enzymes. Thus, we expect that MLL3/4 loss is resulting in the failure to recruit CBP/P300 to those sites. The failure to recruit the acetyltransferases to these sites is expected to have more wide-ranging effects than H3K27 acetylation (e.g. likely H2B acetylation). We thank the reviewer for making us aware of the H2B paper, which we now reference in our text (line 525-526). We also try to better clarify distinction between the published H3K27 mutant and our work (line 525-526).*

*Reviewer 2 says: “A number of studies in recent years have suggested that this view is perhaps an oversimplification. Most relevant to this work are two studies from 2017 (PMID 28967912 and 28483418). Two other studies, PMID 29313530 and 29255264, are also relevant but not cited.*

*In terms of the two 2017 papers, these are the same references brought up by reviewer 1. Again, these two papers are focusing on the catalytic mutants, not the loss of the proteins themselves. Please see our response above. We are also aware of the other excellent papers by Bing Ren and colleagues that this reviewer brings up: PMID 29313530 and 29255264. These papers focus on other roles of MLL3/4 derived H3K4me1 including chromatin looping and BAF complex recruitment respectively. While we did not look at these events, we agree that these papers are relevant and should have been included (line 528-531). In particular, we find it remarkable that even with all these events (P300 recruitment, looping, and BAF recruitment) potentially lost at many sites, there is relatively little impact on the gain in formative gene expression during the transition. We have added these citations and this point to the discussion.*

Specific Points from Reviewer #1:

The authors compare Mll3KO, Mll3/4 DKO and WT with each other. It is well described how the single and the double KO are related to each other, however, it is unclear how the WT relates to the KO strains. Is it the parental strain for the single KO? This should be clarified.

*Reply: We have clarified their relationships in the methods (line 576-578). In short, the derivation of the original MLL3KO/MLL4fl lines are from a mix of C57BL/6J and 129 backgrounds (Kai Ge, Personal Communication). The WT and dCDs in our manuscript are R1 ESCs which are 129 background.*

*Given the different strain backgrounds, we now add the transcriptional comparison and differential gene expression analysis of naive/formative MLL3KO vs. DKO (Fig.S1F,G). We find a slight reduction in the total number of differential genes when comparing MLL3KO vs DKO relative to WT vs. DKO. This comparison further reduced the number of down differential genes in both the naive and formative states by ~100 genes. E.g. down formative genes went from 461 (WT vs DKO) to 387 (MLL3KO vs DKO), strengthening our previous claim of a surprisingly minor phenotype on formative transcription.*

Fig S1B: Labelling issue: in the text it says that the DKO grow slower, but the figure shows that the Mll3KO grows slower compared to the W or the DKO. Which one is correct?

*Reply: We thank the reviewer for catching labeling error in this figure. To be 100% sure, we have updated the figure with newer data including 4 independent replicates. Our claim that DKOs proliferate slower is unchanged (line 140).*

Similar to the previous comment: the color coding on the supplemental figures is not always clear: the DKO is sometimes orange and sometimes pink.

*Reply: We have made consistent the coloring for the growth assay panels.*

Figure 2H and after: what does the "Others" mean and how were they separated from "promoters" and "Distal elements"

*Reply: Feature assignment by Homer includes UTR regions, exons, and transcriptional termination sites. These and unassignable/unannotated features comprise the “Others” category. We have clarified this in the legends (lines 982 and 997) and methods (lines 768-771).*

Figure 2I and 3F: the dependent should be removed from the figure, the authors show both, dependent and independent modifications.

*Reply: Fixed.*

Figure 6A /B/C: it is unclear how the numbers of enhancers related here to each other: in Figure A the authors identify 251 dependent genes, in Figure 6B this number changes to 215 and in Figure 6C to 131. How are these numbers related to each other?

*Reply: We thank the reviewer for pointing this out. It should be 215 and we have corrected this typo throughout the manuscript.*

*Fig.6C represents only those genes (131 of 215) which we can informatically associate with a nearby enhancer. We have better clarified this in the figure legend (lines 1047-1048).*

Figure S2A: Labelling issue: in the main text(line number 183), it should be fig.S2A, but labeled as fig.S1A.

*Reply: Fixed.*

Figure S3B, the citation of Buecker et al.2017, should be Buecker et al.2014.

*Reply: Fixed.*

For figure 3I, the description and discussion of this figure from line 254 to 259 are not very clear in the main text. Please polish this part.

*Reply: We have better clarified this section about Fig.3I in the main text (lines 263-268).*

Figure 5E, the information of the metric is too short and is not mentioned in the method part. and please add more detailed discription of Figure 5E and F.

*Reply: Per reviewer #2’s comment on our metric we have removed these panels and replaced with panels looking at total number of dependent enhancers and proportion of impacted enhancers from the supplementals.*

Specific points from reviewer #2

1. Regarding the growth / morphology of the DKO ESCs, the authors minimize the growth / morphology defects of the DKO (lines 127-136). I would say that the morphology differences as shown are substantial, given that the cells are cultured in 2i. The DKO colony shown is very much different from the WT and MLL3KO. Also, the authors state that the DKO grow at a "slightly slower rate" shown in Fig. S1B. The labels on that figure indicate that MLL3KO are the slowest growing cells. Also, the growth rate of the cells labeled in green seems to be significantly different than that of the other two cell lines, so I don't think it's fair to minimize this as a "slightly slower rate". Related. The "similar levels of OCT4 and NANOG" by WB actually do look lower in the DKO cells (hard to tell / quantify with this assay), which would be in line with the reduced pluripotency evidenced by morphology.

*Reply: See response to reviewer #1 about growth assays figure. In short, yes DKOs have a proliferation phenotype relative to MLL3KO counterparts in our hands. We found this proliferation defect reproduced in multiple DKO clones in addition to the one we used for downstream study. We agree the language we used may be too soft relative to the phenotype. We have made changes accordingly (lines 140-146). This phenotype, if anything, makes it even more surprising that formative transcription can still be normally activated. We suspect the proliferation phenotype is transcription independent. However, not knowing the underlying basis for the slowed proliferation, we want to be cautious of making any strong claims and prefer to leave it as an observation for now.*

*We added quantifications to the OCT4/NANOG western panel. When normalized by TUBULIN levels of NANOG do not appear strongly impacted by loss of MLL3/4. In agreement with your observation, we do find an impact on OCT4 protein e.g. a ratio of 0.59 compared to naïve WT. Our claim is specifically that they exhibit a pluripotent-like identity including expression of Oct4 and Nanog and the ability to self-renew long term. We have emphasized this more clearly in the text (line 145).*

2. I disagree with the interpretation that "gene regulation is largely maintained in the absence of MLL3/4", with a "slight dampening of gene expression" and a "relatively normal" transition from naïve to formative in the DKO - lines 145-152. By eye, it seems like the MLL3/4 DKO both fail to upregulate formative genes and downregulate naïve genes to the same extent observed from the WT, by about 4-fold. This isn't necessarily an inconsequential effect on gene expression, particularly when considering the scope of the defects as shown in Fig. 1G.

*Reply: We agree that the slope (i.e. dynamic range) is reduced, even though the correlation and directionality suggest a surprisingly normal overall transition to the formative state. This reduction in slope is largely driven by a reduction in expression of naïve enriched genes in the naïve state as seen in our new Fig.S1D. We agree this an interesting observation and is consistent with our later analyses such as Fig.3I and Fig.5B (states 1 and 15), where we also point this observation out. We do not believe this observation alters the conclusion that the formative program is still largely activated upon differentiation, again clear in Fig.S1D. We now further clarify this in the text (lines 153-158).*

3. It would be helpful to define the metrics for differential expression in the text in paragraph starting on line 153. Also, in this analysis, I'd like to see box plot or comparative density plot for expression of naïve enriched, formative enriched, and unchanged in WT and DKO cells under both naïve and formative culture conditions.

*Reply: We have added the DGE criteria in the text. We also added a supplemental figure (Fig. S1D) with a boxplot for the different gene groups for WT, MLL3KO, and DKO lines and with brief descriptions in the text (line 156). We thank the reviewer’s suggestion as it does help explain the change in slope as mentioned above in point 2.*

4. The authors state the purpose of determining whether gene expression changes were due to direct or indirect effects of MLL3/4 loss (line 169-170) and proceed to describe H3K4me1 genomics. If they want to determine effects of HMT loss, it would be better to focus this analysis based on MLL3/4 enrichment.

*Reply: In our hands, the commercially available antibodies for MLL3 and MLL4 have high background which necessitates subtraction with knockout controls resulting in poor sensitivity. We believe this issue diminishes the utility of MLL3/4 peaks to assess direct function. Therefore, we use H3K4me1 as a surrogate as in our hands H3K4me1 antibodies produce very robust signal. Consistent with the validity of the approach, we do see good overall correlation of MLL3/4 signal with sites requiring MLL3/4 for H3K4me1, even though the sensitivity of MLL3/4 signal is dramatically lower. We include data with dCD cells as an important complementary piece of evidence for the validity of this approach. We now mention these issues in the revised methods (lines 697 and 757).*

5. Line 187 - the authors report few transcriptional differences between the catalytic dead mutants and wild type cells. When coupled with the larger transcriptional disruptions observed in the DKO cells, this finding suggests that loss of the MLL3/4 proteins is more detrimental than loss of catalytic activity. This is the conclusion reached previously by Wysocka and Shilatifard.

*Reply: Yes, it has been shown that MLL3/4 catalytic activity is not required in steady state naive culture conditions. Here we also show this in a cell state transition/differentiation. Since this paper went out for review we have become aware of a preprint that focuses on MLL3/4 catalytic activity in cell differentiation (https://www.biorxiv.org/content/10.1101/2020.09.14.296558v2). We now cite that as well as described at the beginning of this response. We note that the catalytic dead results are not a major focus of our study nor meant to be where the novelty lies.*

6. The authors are reporting that ~2/3 of the H3K4me1 sites of enrichment are independent of MLL3/4. Does this agree with western blot or proteomics analysis of H3K4me1 levels in the DKO and catalytic dead mutant cell lines? Does this agree with previous data sets published by either Wysocka or Shilatifard?

*Reply: Indeed, this agrees with previous studies including mass spectrometry indicating ~50% remaining H3K4me1 in naive MLL3 and MLL4 double knockout cells from Dorighi et al. 2017. We only lose ~1/3 the total number of H3K4me1 sites. We performed westerns on acid-extracted histones for H3K4me1 with quantifications (Fig.S2B). Our data is consistent with previous data for double knockouts and catalytic dead mutants in the naive state with a loss of ~50% H3K4me1 and also shows a similar impact to H3K4me1 in formative state. We have modified the text accordingly (lines 200-203).*

7. The authors report that MLL3/4 loss has no effect on transcription of the dependent naïve peaks. This is different than the conclusion reached by Wysocka, where MLL3/4 loss had a distance-dependent effect on nearest neighboring genes. Can the authors explain the discrepancy?

*Reply: Our expression data also shows a reduction in expression from TSSs nearby MLL3/4 peaks (Fig. S2g). However, when we break it down into naïve only vs shared MLL3/4 dependent H3K4me1 peaks, we do not see a significance difference at the naïve specific peaks, but do so at the shared peaks (Figs. 2K and S4B). Also, we see a significant difference when we focus on expression TSSs near MLL3/4 dependent H3K4me/H3K27ac peaks (State 15 on Fig.5B and Fig.S6A). Therefore, we do not believe there is a discrepancy. It just represents a deeper analysis that we were able to do given that we have both naïve and formative data allowing us to subset into naïve specific, shared, and formative specific. We try to add some language to explain the differences between the naïve data between our and the Wysocka paper without distracting from our larger focus on activation of the formative expression program where we see very little impact of MLL3/4 loss (lines 225-229).*

8. Line 201 - authors state that MLL3/4 ChIP-seq data show enrichment at H3K4me1 sites whether naïve-enriched or shared, but based on the average profile the level of enrichment is much lower at the shared group, and based on the heat map, many of the shared peaks do not appear enriched for MLL3/4. This makes me wonder whether these non-MLL3/4 bound H3K4me1 peaks are real (and therefore should be detectable in antibody-independent proteomics assays, see point 6 above) or whether they are some noise inherent to H3K4me1 genomics.

*Reply: We break the shared group into independent and dependent groups (Fig.2J dark grey vs light grey cluster respectively). MLL3/4 ChIP-seq has the highest enrichment at shared dependent peaks and second highest at naïve-enriched peaks. Is the H3K4me1 signal in the MLL3/4 independent cluster real? Based on the remaining proportion of H3K4me1 by western and previous proteomics we believe so.*

9. What enzyme is responsible for non-MLL3/4 H3K4me1?

*Reply: We attempted to address your question with published ChIP-seq data for MLL1 and MLL2 as they appear to be strong candidates. We were unable to find published LIF+2i data, but did find ChIP-seq from LIF culture conditions for MLL1 (Zhang 2019) and MLL2 (Hu 2018). We processed the data with proper controls (subtracting input from MLL1/Zhang and subtracting shMLL2 knockdown ChIPseq from MLL2/Hu) and found some enrichment of both MLL1 or MLL2 at MLL3/4 independent sites relative to background and the MLL3/4 dependent sites. However, enrichment at promoters (right two panels) was much stronger (4x and 10x for MLL1 and MLL2 respectively). Because of the culture conditions and the fact enrichment at these distal sites is not very strong we believe it remains unclear whether MLL1/2 is the source of the remaining H3K4me1 sites. Therefore, we have decided not to include this data. For now, we hope our speculation about MLL1/2 and other SET domain containing candidates in the discussion is sufficient.*

*(Please see supplementary information - response to reviewers for figure)*

10. Line 223 - the authors state that they intersected H3K27ac C&T peaks with ATAC peaks to enrich for active enhancers. One - wouldn't this also include promoters, or were they computationally excluded? Two - why didn't they perform this additional filter with the H3K4me1 data to identify active regulatory elements? Given the spread or breadth of H3K4me1 peaks, such analysis might change the interpretation of effects on nearest neighboring gene expression shown in the first two figures.

*Reply: You are correct promoters are computationally excluded in order to focus on distal sites. We now state this more clearly in the text (line 241 and 383).*

*We originally did not subset H3K4me1 by ATAC because this modification tends to mark larger domains and we were unsure about how well accessibility correlates with primed (H3K4me1+ only) enhancers. Based on your suggestion, we performed nearest neighbor analysis again considering H3K4me1 now subset by ATAC (Fig.S4B, lines 285-287). It did not change the conclusion that we originally reached from Fig.2K using all H3K4me1 sites.*

11. Regarding Figure 3, have the authors done similar experiments in the catalytic dead cells to determine whether the MLL3/4-dependent regions require enzymatic activity or simply the protein itself for proper H3K27ac?

*Reply: We considered these experiments but remained focused on the protein knockouts for clarity of the study about MLL3/4 independent sites. Because Dorighi et al. show convincingly that H3K27ac at most sites is unaffected by loss of catalytic activity we would expect similar behavior in our case.*

12. Line 347 - I find this conclusion confusing, that "only a small number of TFs is dependent on MLL3/4 for maintaining and/or establishing cell state specific H3K4me1 independent of H3K27ac" based on motif analysis only.

*Reply: We have changed this sentence (line 366) to instead suggest that a subset of TFs require MLL3/4 to maintain and or establish H3K27ac. In the discussion we elaborate. We are trying to make the point that this data suggests that different TFs have different requirements for MLL3/4 in the activation of enhancers.*

13. Line 375 RE enhancer redundancy. The authors report a method by which they attempt to normalize for potential enhancer redundancy. I am not certain that this method (multiplying the number of nearby enhancers for each gene by the number of MLL3/4 dependent acetylation sites) is meaningful in the way that the authors would like it to be.

*Reply: We have replaced these panels with panels that were in the supplemental figures measuring total numbers of impacted enhancers and proportion of enhancers lost.*

14. The overall conclusion that I get is that MLL3/4 is important for maintaining cell state but is not important for cell state transitions. This is in contrast to work from the Ge lab and should be discussed.

*Reply: We believe our work to be consistent with that from the Ge Lab. In the naive case DKO has a transcriptional phenotype which appears to be at least partially alleviated when differentiated into the formative state. While in adipogenesis and myogenesis models, the Ge Lab shows differentiation is catastrophically affected by loss of MLL3/4. The differences between our studies are important because it demonstrates a cell identity/context specific function of MLL3/4 e.g. it is not an essential factor generalizable to all differentiations. We believe that a direct reason for this is the compendium of TFs that require or do not require MLL3/4 is different for each transition. For instance, PPARy and CREB-a, are major adipogenic factors which Ge Lab has shown are MLL3/4 dependent, while at least some of the pluripotency factors and the drivers of formative state are likely to be MLL3/4 independent TFs in our context. This is supported by our finding of enhancer activation for key formative markers in the absence of MLL3/4. This discovery is interesting because it may shed light on why some tissues are selectively vulnerable to MLL3/4 mutations in normal development and in abnormal development such as cancer. Likewise, whether there is a switch in gene regulation by a different complex in early development to MLL3/4 later in lineage specification is an intriguing avenue of further study.*

**Second round of review**

**Reviewer 1**

The authors have adequately incoporarted suggested changes and addressed previous concerns. There are no more outstanding issues and I congratulate the authors on a beautiful paper.

**Reviewer 2**

The authors have addressed most of my concerns and I have no additional comments.

**Authors Response**

**Point-by-point responses to the reviewers’ comments:**

Reviewer #1:

The authors have adequately incoporarted suggested changes and addressed previous concerns. There are no more outstanding issues and I congratulate the authors on a beautiful paper.

Reviewer #2:

The authors have addressed most of my concerns and I have no additional comments.

*Reply: We thank the reviewers for recommending publication of paper as is.*
